# Nuclear Radiation Degradation Study on HD Camera Based on CMOS Image Sensor at Different Dose Rates

**DOI:** 10.3390/s18020514

**Published:** 2018-02-08

**Authors:** Congzheng Wang, Song Hu, Chunming Gao, Chang Feng

**Affiliations:** 1 Institute of Optics and Electronics, Chinese Academy of Sciences, No. 1 Guangdian Avenue Xihang Port, Shuangliu, Chengdu 610209, China; husong@ioe.ac.cn (S.H.); fc407@ioe.ac.cn (C.F.); 2 School of Optoelectronic Information, University of Electronic Science and Technology of China, No. 4, Block 2, North Jianshe Road, Chengdu 610054, China; gaocm@uestc.edu.cn; 3 University of Chinese Academy of Sciences, No. 19, Yuquan Road, Shijingshan District, Beijing 100049, China

**Keywords:** CMOS image sensor, radiation damage, dose rate, HD camera

## Abstract

In this work, we irradiated a high-definition (HD) industrial camera based on a commercial-off-the-shelf (COTS) CMOS image sensor (CIS) with Cobalt-60 gamma-rays. All components of the camera under test were fabricated without radiation hardening, except for the lens. The irradiation experiments of the HD camera under biased conditions were carried out at 1.0, 10.0, 20.0, 50.0 and 100.0 Gy/h. During the experiment, we found that the tested camera showed a remarkable degradation after irradiation and differed in the dose rates. With the increase of dose rate, the same target images become brighter. Under the same dose rate, the radiation effect in bright area is lower than that in dark area. Under different dose rates, the higher the dose rate is, the worse the radiation effect will be in both bright and dark areas. And the standard deviations of bright and dark areas become greater. Furthermore, through the progressive degradation analysis of the captured image, experimental results demonstrate that the attenuation of signal to noise ratio (SNR) versus radiation time is not obvious at the same dose rate, and the degradation is more and more serious with increasing dose rate. Additionally, the decrease rate of SNR at 20.0, 50.0 and 100.0 Gy/h is far greater than that at 1.0 and 10.0 Gy/h. Even so, we confirm that the HD industrial camera is still working at 10.0 Gy/h during the 8 h of measurements, with a moderate decrease of the SNR (5 dB). The work is valuable and can provide suggestion for camera users in the radiation field.

## 1. Introduction

CMOS image sensors (CISs) have evolved rapidly in recent years, taking advantage of the reduction in the transistor size. Now, CISs have many advantages, such as low power consumption, wide dynamic range, low noise, high frame frequency, high data output rate, and simple control timing sequence [1,2,3,4]. CISs, with radiation hardening (RH-CIS) in particular, have been widely used for video inspection during outages of nuclear plant reactors, and in space detector applications in satellites owing to their generally higher radiation tolerance compared to common CISs [5,6,7]. However, common CISs have lower cost, higher spatial resolution, lower dark current and higher reliability, and nuclear radiation damage is acceptable in the specific low dose rate environment [8,9,10,11]. Analysis of nuclear radiation degradation of common CMOS camera can provide important evidence for design and selection of camera applied in radiation field. Therefore, it is necessary to analyze nuclear radiation degradation of common CMOS camera.

The radiation damage mainly includes total ionizing dose (TID) damage at different dose rates, displacement dose (DD) damage, and single-event transient (SET) damage. TID damage induces the performance degradation and even causes functional failure of CISs, so this damage is one of the most concerning with the application of HD CMOS camera. Goiffon et al. have presented TID versus DD damage in the CISs induced by proton radiation [12]. Leonello Servoli et al. have studied the progressive damaging of the CISs without radiation hardening [13]. Zujun Wang et al. have reported the degradation of 3T CISs manufactured in 0.35-μm technology induced by TID irradiation at 7.2 and 1800.0 Gy(Si)/h and unbiased conditions [14]. Although several articles have investigated TID-induced degradation on CISs, few papers have focused on the effect of dose rate on the image quality of HD camera based on CISs.

The aim of this work is to confirm the performance degradation on HD CMOS camera at various dose rates. Firstly, the degradation of the image is observed and recorded by radiation experiments. Then, based on data analysis using MATLAB, we investigate the degradation results and process of the tested camera induced by TID damage, and the SNR versus the dose rates is reported. Finally, application suggestions of the HD CMOS camera are presented by intuitive analysis of the output data of the camera for the users of the camera in the radiation field.

## 2. The HD Industrial Camera

**Figure 1:**
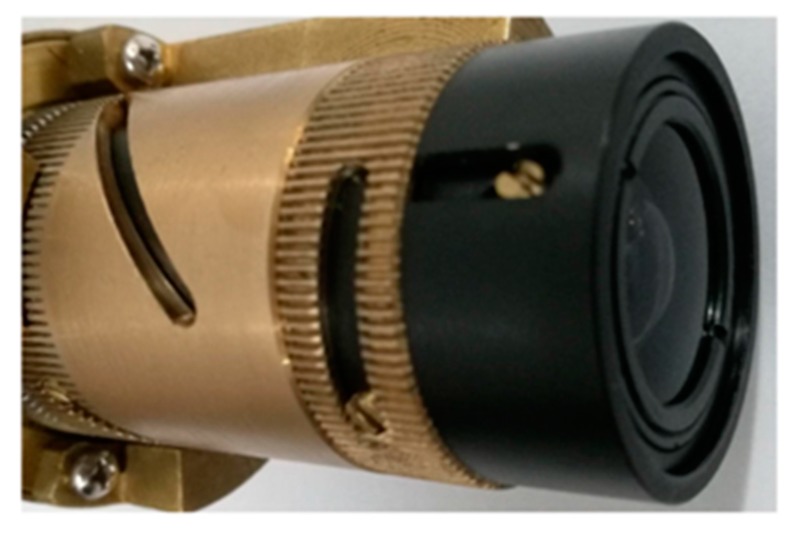
Lens for the test.

The industrial camera under test mainly consists of a non-browning zoom lens, a board-level camera module, data acquisition unit, and system software. All parts of the camera are housed in an ordinary aluminum alloy shell. The lens is available in the camera module based on 2/3” progressive scan CIS and supports C-mount, as is shown in Figure 1. To avoid the impact of the lens in the test, the selected lens was highly resistant to radiation. Additionally, it was made from ZF7 optical glass, which is radiation hard material; that is, browning or discoloration will not occur when the lens is exposed to radiation. The thickness of the glass is about 62 mm, which is determined by the length of the lens. The radiation resistance of the lens ensures that its attenuation is not obvious during the measurements, and the effect of the attenuation on the experiment can be ignored.

**Figure 2:**
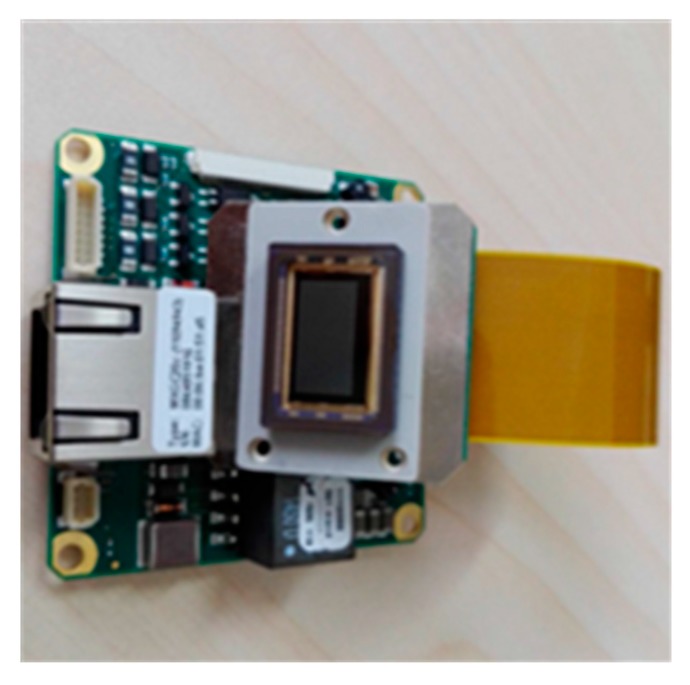
HD camera module under test.

Figure 2 shows that the HD camera module is mainly made up of CIS, FPGA control chip, and communication unit. The CIS is the imaging component of the camera, and can capture degradation process of camera. The CMV2000 manufactured in the standard 0.18-μm CIS technology from CMOSIS Company is selected in this paper because of its high sensitivity, low noise, and high frame. The image array of the CIS is made up of 2048 × 1088 pixels with size of 5.5 μm × 5.5 μm. The pixel has state-of-the-art architecture, which offers correlated double sampling (CDS) based on a 4T pinned photodiode front end, reducing the fixed pattern noise and dark noise significantly. The pixel is controlled by 8T pipelined global shutter, which allows exposure during reading out to improve the frame frequency. FPGA control chip and communication unit are also irradiated with gamma rays. It has been proved that Flash-based FPGA with a 20% reduction in working frequency can work normally during the TID of 800 Gy. The FPGA was used to realize the timing drive and digital signal reading of the image sensor.

## 3. Experimental Details

Five HD cameras are irradiated by γ photons produced by Cobalt-60 gamma-ray source (at China Isotope & Radiation Corporation, Chengdu, China) at room temperature. The dose rate of test position is calibrated by the Silver Dichromate Dosimeter before the irradiation test. The cameras have serial numbers from A01 to A05, and all the CISs come from the same batch. The cameras are irradiated at 1.0, 10.0, 20.0, 50.0 and 100.0 Gy/h for 8 h. This is because the camera at 100.0 Gy/h cannot survive after more than 8 h; that is, there is no image output after this period of time. The tests for every camera took the same amount of time. The irradiation experiment conditions and the serial number of the tested cameras are presented in Table 1. All cameras are supplied with 12 V DC, and can output real-time video signal.

The test method used in this paper is as follows: First, an omnidirectional light source is used to ensure that the light of the test field is enough. The switch of the light source can be controlled to measure the dark of the sensor. Then, the data of the five cameras are continuously recorded into a video stream during the progressive damaging, respectively. To compare the SNR of the cameras before, during and after radiation, the video stream includes images under the three conditions. Finally, the degradation level of the camera is calculated as the European Machine Vision Association (EMVA) 1288 standard.

## 4. Data Analysis

**Figure 3:**
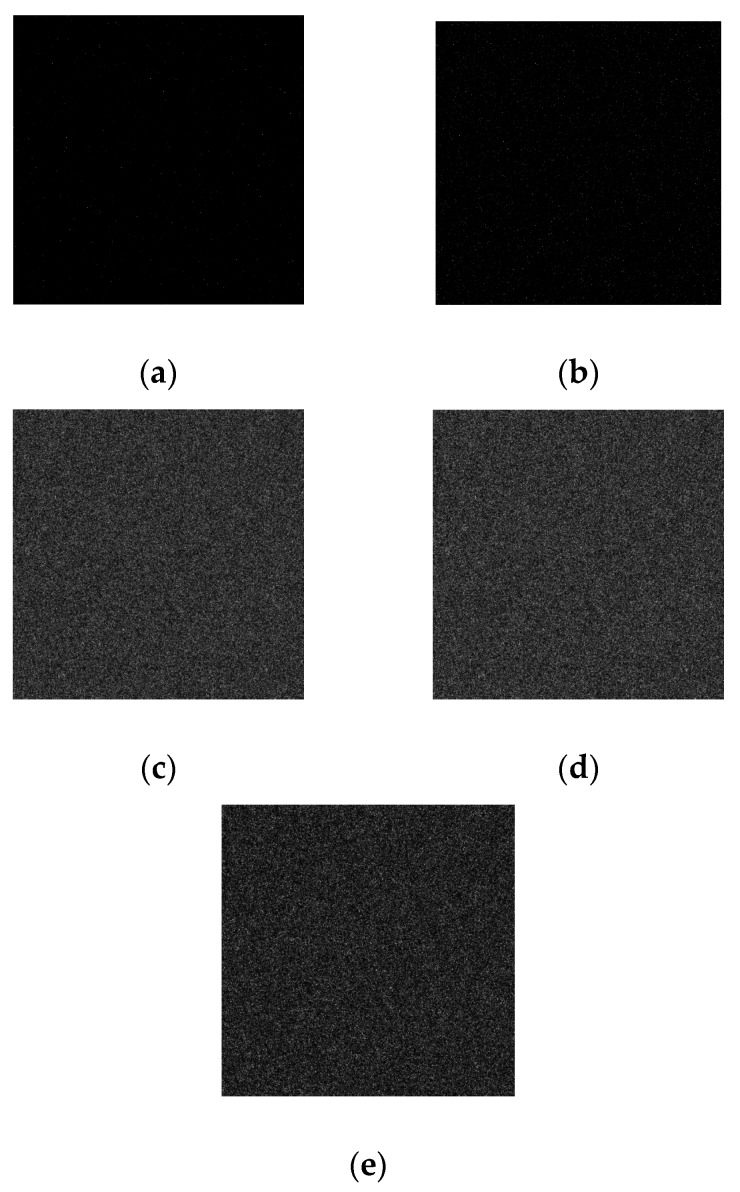
Images acquired without light source under the condition of five dose rates, respectively. (**a**) Image acquired at 1.0 Gy/h. (**b**) Image acquired at 10.0 Gy/h. (**c**) Image acquired at 20.0 Gy/h. (**d**) Image acquired at 50.0 Gy/h. (**e**) Image acquired at 100.0 Gy/h.

**Figure 4:**
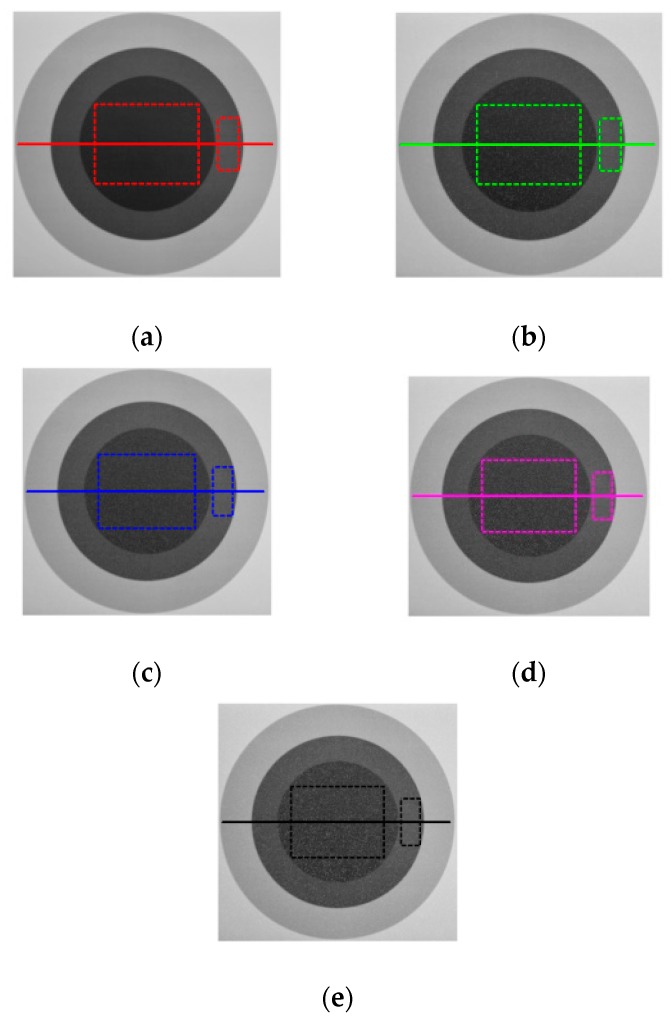
Images acquired with light source under the condition of five dose rates, respectively. (**a**) Image acquired at 1.0 Gy/h. (**b**) Image acquired at 10.0 Gy/h. (**c**) Image acquired at 20.0 Gy/h. (**d**) Image acquired at 50.0 Gy/h. (**e**) Image acquired at 100.0 Gy/h.

The dose rate is a constant value for every camera within a certain spatial range during the irradiation test. To avoid the influence of TID on the analysis of dose rate, before the irradiation test, the light source is shut down. At the beginning of the irradiation test, all online frames are captured at five different dose rates to measure the darks at the same time, respectively. The corresponding images can be seen in Figure 3a–e. Then, the light source is immediately turned on. The images are also grabbed at five different dose rates, respectively. There are relatively bright and dark areas with homogeneous background in the viewing field of the camera. The grabbed images can be seen in Figure 4a–e.

**Figure 5:**
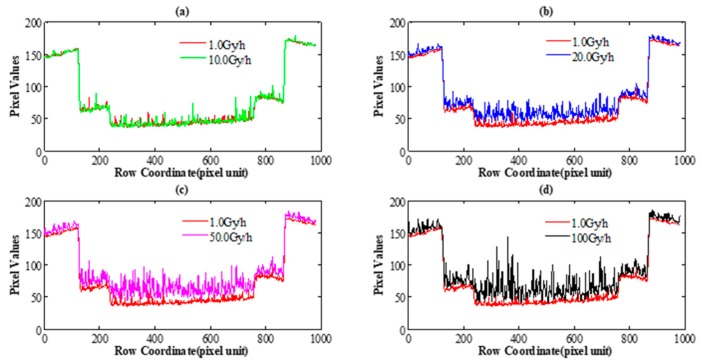
(**a**) The comparison of horizontal cross-section in Figure 4a (red solid line) and Figure 4b (green solid line). (**b**) The comparison of horizontal cross-section in Figure 4a (red solid line) and Figure 4c (blue solid line). (**c**) The comparison of horizontal cross section in Figure 4a (red solid line) and Figure 4d (pink solid line). (**d**) The comparison of horizontal cross-section in Figure 4a (red solid line) and Figure 4e (black solid line).

One dark disc and two relatively bright rings correspond to the object pixel in the viewing field, and the white dots are the protons detected by the camera. In order to intuitively evaluate the radiation effect on the object at different dose rates, the horizontal cross-section of the pixel values in Figure 4a–e are calculated. The corresponding comparison of the horizontal cross-section of the pixel values is shown in Figure 5a–d, respectively. One visible effect of nuclear radiation damage is that the brighter the pixel is, the lower the radiation effect is at the same dose rate, and vice versa. The results also show that with the increase of dose rate, overall grey values in the image grow; namely, the same target images become brighter.

**Figure 6:**
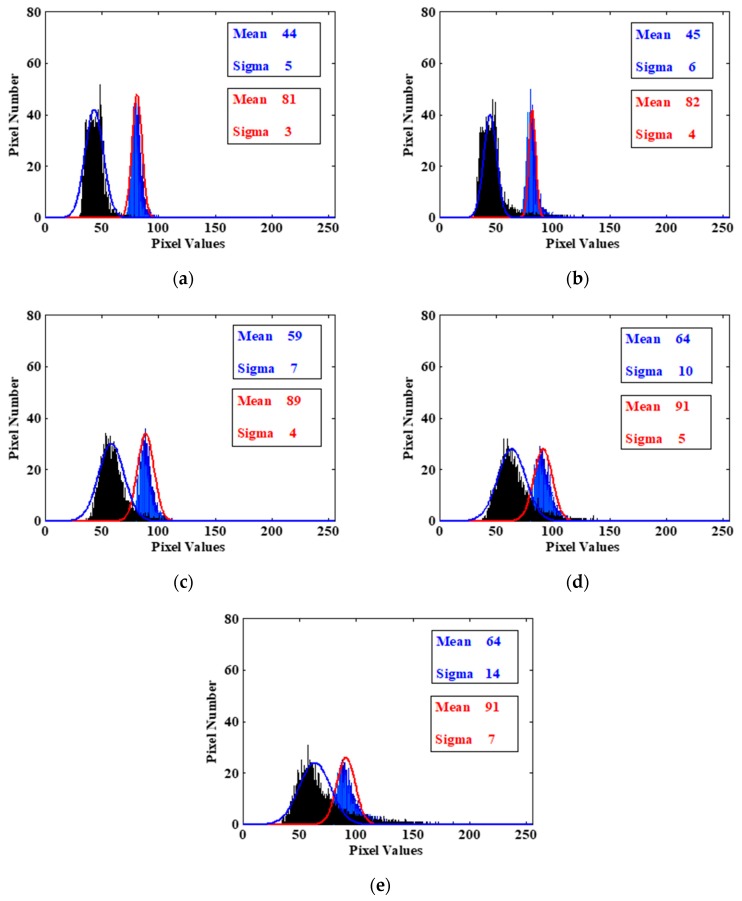
(**a**) The histogram comparison of the dark and bright rectangle areas in Figure 4a. (**b**) The histogram comparison of the dark and bright rectangle areas in Figure 4b. (**c**) The histogram comparison of the dark and bright rectangle areas in Figure 4c. (**d**) The histogram comparison of the dark and bright rectangle areas in Figure 4d. (**e**) The histogram comparison of the dark and bright rectangle areas in Figure 4e.

To study such an effect in detail, we employ image histograms to analyze noise distribution. The big rectangular box corresponds to the dark area, and the small one corresponds to the bright area in Figure 4a–e. Using the image histogram, the analysis results at five different dose rates are shown in Figure 6, respectively. According to Figure 6, we can draw the following conclusions: At the same dose rate, compared with the dark area, the radiation effect in the bright area is lower, and the corresponding variance is smaller. Under different dose rates, the higher the dose rate is, the worse the radiation effect in both bright and dark regions, and the corresponding variances become greater. It is worth noting that with the increase of dose rate, the mean of the two areas also becomes greater, respectively. It indicates that images become brighter. This phenomenon agrees well with the analysis results in Figure 5. It means that the dynamic range of the image decreases, namely, the dynamic characteristics of the system go badly. The results also show that the higher the dose rate is, the larger the noise variance is in the radiation area, namely, the image quality decreases with the increase of radiation noise. This phenomenon agrees well with the visual observation in Figure 4.

## 5. Continuous Degradation Analysis

The continuous data acquisition during the irradiation procedure has been used to record the progressive deterioration of the camera under working conditions. SNR stands for the quality of output signal in a camera based on CIS. As mixed-signal circuitry, the SNR is a key parameter of a camera. The SNR can be given as:

(1) $$\mathrm{SNR}=20\mathrm{lg}\left(\frac{V_{\mathrm{Sig}}}{V_{N}}\right)$$

where $V_{\mathrm{Sig}}$ stands for the signal output, $V_{N}$ stands for the whole noise at a given signal level.

According to the EMVA1288 standard, first, we define the mean gray value of bright and dark rectangle area in the captured image, respectively:

(2) $$\{\begin{array}{c} \mu_{y,\mathrm{bright}}=\frac{1}{\mathrm{MN}}\overset{M-1}{\underset{m=0}{\sum }}\overset{N-1}{\underset{n=0}{\sum }}y_{\mathrm{bright}}\left[m\right]\left[n\right]\\ \mu_{y,\mathrm{dark}}=\frac{1}{\mathrm{MN}}\overset{M-1}{\underset{m=0}{\sum }}\overset{N-1}{\underset{n=0}{\sum }}y_{\mathrm{dark}}\left[m\right]\left[n\right] \end{array}$$

where M and N are the number of rows and columns, respectively, of the bright rectangular area $y_{\mathrm{bright}}$ and the dark rectangle area $y_{\mathrm{dark}}$. m and n are the row and column indices of the array, respectively.

Then, the SNRs of the bright and dark rectangle area are given as [15]:

(3) $$\{\begin{array}{c} {\mathrm{SNR}}_{\mathrm{bright}}=20\mathrm{lg}\left(\frac{\mu_{y,\mathrm{bright}}-\mu_{y,D}}{\sigma_{y,\mathrm{bright}}}\right)\\ {\mathrm{SNR}}_{\mathrm{dark}}=20\mathrm{lg}\left(\frac{\mu_{y,\mathrm{dark}}-\mu_{y,D}}{\sigma_{y,\mathrm{dark}}}\right) \end{array}$$

where $\mu_{y,D}$ is the mean gray value in the captured corresponding image without light source, $\sigma_{y,\mathrm{bright}}$ and $\sigma_{y,\mathrm{dark}}$ are the standard deviation of the gray value of the bright and dark rectangle area, respectively.

**Figure 7:**
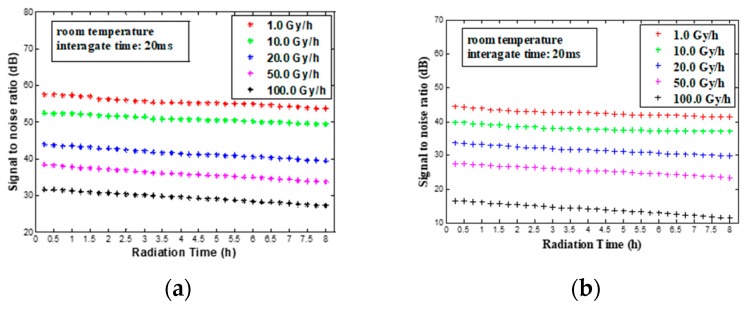
(**a**) ${\mathrm{SNR}}_{\mathrm{bright}}$ versus Radiation time at various dose rates. (**b**) ${\mathrm{SNR}}_{\mathrm{dark}}$ versus Radiation time at various dose rates.

Because the SNR is related to the degree of saturation of the sensors, we measured the ${\mathrm{SNR}}_{\mathrm{bright}}$ and ${\mathrm{SNR}}_{\mathrm{dark}}$ at the same integration time (20 ms) and the same light intensity in this paper. The ${\mathrm{SNR}}_{\mathrm{bright}}$ and ${\mathrm{SNR}}_{\mathrm{dark}}$ at the different dose rates can be seen in Figure 7. According to Figure 7, we can draw the conclusion that the attenuation of both ${\mathrm{SNR}}_{\mathrm{bright}}$ and ${\mathrm{SNR}}_{\mathrm{dark}}$ are not obvious with increasing radiation time under the condition of the same dose rate during the 8 h of measurements. Meanwhile, ${\mathrm{SNR}}_{\mathrm{bright}}$ is much better than ${\mathrm{SNR}}_{\mathrm{dark}}$ at the same dose rate. This phenomenon agrees well with the analysis results in Figure 6. And the decrease of both ${\mathrm{SNR}}_{\mathrm{bright}}$ and ${\mathrm{SNR}}_{\mathrm{dark}}$ varies with the dose rates as shown in Figure 7. ${\mathrm{SNR}}_{\mathrm{bright}}$ and ${\mathrm{SNR}}_{\mathrm{dark}}$ decrease at 20.0, 50.0 and 100.0 Gy/h are far greater than those at 1.0 and 10.0 Gy/h. It is worth mentioning that the decrease of ${\mathrm{SNR}}_{\mathrm{bright}}$ and ${\mathrm{SNR}}_{\mathrm{dark}}$ at 10.0 Gy/h are still mild, and their attenuation is within 5 dB. It is higher than 40 dB, which meets the needs of general monitoring in the image quality.

It is worth noting that the decrease of both ${\mathrm{SNR}}_{\mathrm{bright}}$ and ${\mathrm{SNR}}_{\mathrm{dark}}$ varies with the dose rates in our measurements. This is different from the results under the unbiased condition in reference [14]. It is possible that electron-hole pairs of the pinned photodiode produced by radiation are more difficult to be compounded under the action of an electric field generated by the biased condition. So sensors are much more sensitive to dose rates and TID at biased condition.

## 6. Conclusions

In this article, we have investigated the nuclear radiation degradation on HD CMOS camera under biased conditions at 1.0, 10.0, 20.0, 50.0 and 100.0 Gy/h at room temperature. The CIS manufactured using a standard 0.18-μm CMOS technology with four-Transistor pixel PPD architecture without radiation hardening is the key module of the HD CMOS camera. The camera was acquiring data continuously during the irradiation, allowing for the measurement of the damaging effects under working conditions. The behavior of the tested cameras shows a remarkable degradation after irradiation, and differs with the dose rates.

During irradiation, one visible effect of the camera irradiated by ^60^Co γ rays is that the brighter the pixel is, the lower the radiation effect is at the same dose rate. The higher the dose rate is, the larger the noise variance of the radiation area is at the beginning of the irradiation test. However, the analysis of radiation degradation is not comprehensive enough. The test does not discriminate between TID and dose rate contribution to the radiation effect. Actually, the TID may be the main cause of the noise variance originating in the radiation area. In the future, more radiation experiments of the same TID will be carried out to confirm the conclusion of this paper.

By analyzing the SNR of the image, we obtain that the attenuation of the SNR is not obvious with increasing radiation time, and the SNR decrease varies with the dose rates. Even so, The SNR decrease at 10.0 Gy/h is still mild, and its attenuation is within 5 dB. Hence, a HD industrial camera based on common CIS can be appropriate for some applications where ionizing radiations with moderate radiation damage are involved.

## Figures and Tables

**Table 1:** Irradiation experiment conditions and the serial numbers of the tested cameras.

Camera Number	Bias Condition	Dose Rate (Gy/h)	Total Dose (Gy)
A01	Biased	1.0	8.0
A02	Biased	10.0	80.0
A03	Biased	20.0	160.0
A04	Biased	50.0	400.0
A05	Biased	100.0	800.0
